# Potential of On-Line Visible and Near Infrared Spectroscopy for Measurement of pH for Deriving Variable Rate Lime Recommendations

**DOI:** 10.3390/s130810177

**Published:** 2013-08-08

**Authors:** Yücel Tekin, Boyan Kuang, Abdul M. Mouazen

**Affiliations:** 1 Vocational School of Technical Sciences, Uludag University, Bursa 16059, Turkey; 2 Environmental Science and *Technology* Department, Cranfield University, Bedfordshire, MK43 0AL, UK; E-Mails: b.kuang@cranfield.ac.uk (B.K.); a.mouazen@cranfield.ac.uk (A.M.M.)

**Keywords:** on-line soil sensor, vis-NIR spectroscopy, soil pH, lime recommendation map

## Abstract

This paper aims at exploring the potential of visible and near infrared (vis-NIR) spectroscopy for on-line measurement of soil pH, with the intention to produce variable rate lime recommendation maps. An on-line vis-NIR soil sensor set up to a frame was used in this study. Lime application maps, based on pH predicted by vis-NIR techniques, were compared with maps based on traditional lab-measured pH. The validation of the calibration model using off-line spectra provided excellent prediction accuracy of pH (*R*^2^ = 0.85, RMSEP = 0.18 and RPD = 2.52), as compared to very good accuracy obtained with the on-line measured spectra (*R*^2^ = 0.81, RMSEP = 0.20 and RPD = 2.14). On-line predicted pH of all points (e.g., 2,160) resulted in the largest overall field virtual lime requirement (1.404 t), as compared to those obtained with 16 validation points off-line prediction (0.28 t), on-line prediction (0.14 t) and laboratory reference measurement (0.48 t). The conclusion is that the vis-NIR spectroscopy can be successfully used for the prediction of soil pH and for deriving lime recommendations. The advantage of the on-line sensor over sampling with limited number of samples is that more detailed information about pH can be obtained, which is the reason for a higher but precise calculated lime recommendation rate.

## 1. Introduction

Soil acidity is one of the important properties that affects availability of nutrients, controls the composition and diversity of the microbial community, alters the equilibrium solid phase and impacts plant response [1]. The indicator of acidity is soil pH, which affects the activity of enzymes due to the pH sensitivity of amino acid functional groups that alter conformational and chemical changes of amino acids essential for binding and catalysis [1]. For soils with a pH lower than 7, natural processes (e.g., rainfall, crop growth and especially leaching of calcium in drainage water) and some farming practices (e.g., use of large amounts of nitrogen fertilisers) tend to acidify soil [2]. Acidifying processes can cause soil pH to fall quite quickly. Soil acidity can be corrected by applying lime, which is relatively insoluble, and most recommendations involve ploughing to incorporate and mix the ameliorant into the soil [3]. Lime addition causes a significant improvement in soil properties in a short time by reducing plasticity and eliminating swelling [4]. Liming may affect both mobility of K through the soil profile and availability of K to crops [5]. However, excessive application of lime has been shown to result in phosphorus adsorption and deficiencies of micronutrients like Fe, Mn, Zn, Cu and B [6]. Maintaining the optimum pH level in the topsoil in all parts of the field is important to achieve optimum yields and consistent quality. If soil acidity is not corrected, this can cause large yield losses. Variable rate lime application may be a solution of avoiding under- or over-application of lime. An automated method to quantify the spatial variation in soil pH accurately and cost-effectively will enable the optimisation of lime application variably over the field.

Precision agriculture involves the use of sensor technologies for mapping the spatial variation in several entities, namely: crop yield, crop growth, soil characteristics, and others. The output of these technologies is useful information for variable rate nutrient and pesticide application, irrigation control, tillage, *etc.* Therefore, precision agriculture makes extensive use of sensors in order to identify proper targets and needs of crops for applying locally varying doses of chemicals [7]. Various types of soil sensor technologies are used, but in many cases these are insufficient for the *in situ* monitoring of plant beds conditions, such as the nutrient concentration, soil compaction, and pH, because particle sizes and plant roots in the solution are non-uniform distributed spatially and with depth [8]. Adamchuk *et al.*[9] concluded that with certain field conditions, on-line mapping can significantly increase the accuracy of soil pH maps and therefore increase the potential profitability of variable rate liming. A sensing technology enabling the on-line measurement of soil pH at fine resolution sampling rate is a crucial requirement to implement variable rate liming.

Visible (vis) and near infrared (NIR) spectroscopy is one of the promising techniques with high potential for meeting the *in situ* soil monitoring requirements. The on-line vis-NIR spectroscopy sensing technology has proven in many cases to provide accurate measurement of key soil properties with direct spectral response in the near infrared (NIR) range, such as soil moisture content (MC), clay content, organic carbon (OC) and total nitrogen (TN) [10–12]. However, less successful measurement of soil properties with indirect spectral responses in the NIR range has been reported [13,14]. Among other soil properties, pH was reported to have indirect spectral response in the NIR range [11]. Shibusawa *et al.*[15] reported a real time portable spectrophotometer for pH measurement based on four wavelengths. In a study carried out in the Flemish part of Belgium, Mouazen *et al.*[10] provided reasonable similarity between laboratory measured and on-line vis-NIR predicted pH maps, without providing statistical evaluation of prediction accuracy. González *et al.*[13] discussed the performance on the vis-NIR spectroscopy for the measurement of soil pH under on-line and non-mobile (off-line) measurement conditions. However, authors did not attempt to produce pH maps, neither lime recommendation maps. To our knowledge, none of the above studies has explored the potential of vis-NIR spectroscopy to derive lime recommendation maps based on pH maps produced under on-line measurement conditions.

The aim of this paper is to explore the potential of a vis-NIR on-line sensor to measure soil pH with enhanced accuracy. Lime recommendation maps produced based on on-line measured pH will be evaluated by comparing with lime maps developed with laboratory analysis of soil pH.

## 2. Materials and Methods

### 2.1. On-Line Soil Sensor

**Figure 1.**
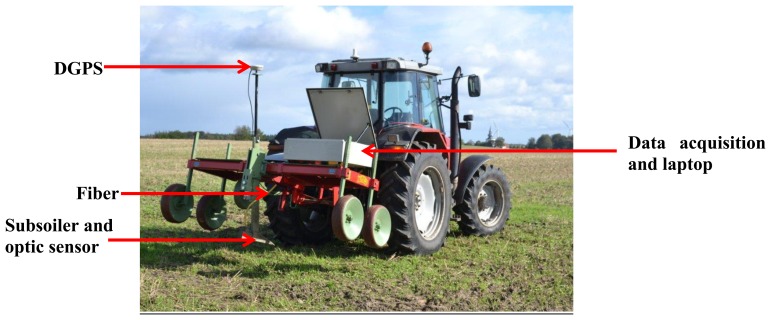
The on-line visible and near infrared (vis-NIR) soil sensor attached to the three point linkage of a tractor [16].

On-line vis-NIR soil sensor (Figure 1) developed by Mouazen [16] was used in this study. It consists of a medium-deep subsoiler, which penetrates the soil to a required depth, making a trench, whose bottom is smoothened by the downwards forces. It was set up to a frame, which was mounted on the three-point linkage of a tractor (Figure 1). In order to measure soil spectra in diffuse reflectance mode, an AgroSpec mobile, fibre type, vis–NIR spectrophotometer (Tec5 Technology for Spectroscopy, Oberursel, Germany) with a measurement range of 305–2,200 nm was used. It is fast, precise and robust, without moving parts, which makes it suitable to be permanently aligned on mobile machines. The light source is a 20 W tungsten halogen lamp illuminating the soil surface. The spectrometer was IP 66 housed, protected for harsh working environments [11]. A differential global positioning system (DGPS; EZ-Guide 250, Trimble, CA, USA) was used to record the position of the on-line measured spectra with sub-meter accuracy. A semi-rugged Panasonic laptop was used for control, data logging and communication. The spectrometer system, laptop and DGPS were powered by the tractor battery.

### 2.2. Experimental Site and On-Line Measurement

**Figure 2.**
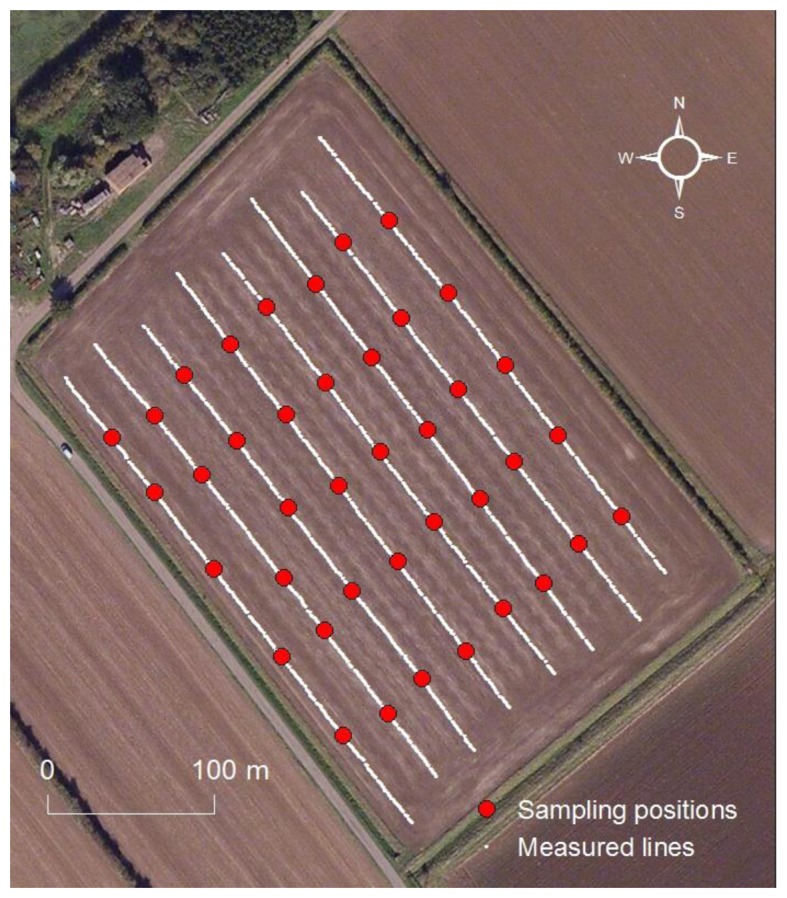
Measured lines with sampling positions, recorded with a DGPS.

One field of 4.17 ha area was considered in this study. It is designated as Ten Acre Meadow field (Table 1) and belongs to the Duck End Farm in Wilstead, Bedfordshire, UK. The field was of clay soil, as shown in Table 1. During the field measurement that took place after crop harvest in the summer of 2012, the on-line vis-NIR sensor was pulled through eight adjacent lines of 20 m intervals at a travel speed of 2 km/h, setting the subsoiler at 15 cm depth. Five soil samples were collected from each transect from the bottom of the trench and the sampling positions were carefully recorded with the DGPS. A total of 40 soil samples were collected during the on-line measurement for calibration and validation (Figure 2).

Each of those samples was equally divided into two parts. One half was used to carry out the laboratory reference measurements of soil pH and particle size distribution (PSD), whereas the other half was used for optical scanning.

### 2.3. Laboratory Analysis

The soil pH was measured by a glass electrode in a 1:5 (volume basis) suspension of soil in a solution of 1 M KCl after shaking on a side-to-side shaker (300 revolutions per minute) for 60 min according to the British Standard (BS) [17]. The PSD was measured by sieving and sedimentation method [18]. The entire sample set of 40 samples were used for pH analysis, whereas only 14 selected samples were used for the PSD analysis.

### 2.4. Off-Line Measurement in the Laboratory

Each soil sample was thoroughly mixed and debris such as plant material and stones were removed. Then each soil sample was placed into three Petri dishes, which were 2 cm in depth and 2 cm in diameter [11]. Before scanning, the soil in a cup was gently pressed and carefully levelled with a spatula to form a smooth scanning surface and increase signal-to-noise ratio, since a smooth soil surface ensures maximum diffuse light reflection captured by the spectrophotometer [19]. The soil samples were scanned using the same mobile, fibre type, vis-NIR spectrophotometer (AgroSpec from tec5), used during the on-line measurement. A 100% white reference was measured before soil scanning, and was repeated every 30 min. A total of 10 scans were collected from each cup, and these were averaged in one spectrum. The three average spectra of each cup were finally averaged in one final spectrum to be used for further analysis.

### 2.5. Model Establishment and Validation

Out of the 40 soil samples collected from Ten Acre Meadow field during the on-line measurement, 24 samples were pooled together in one matrix with other 270 samples, collected previously from several European countries. These 24 samples were added to introduce soil variability of the studied field into the original data set. The decision was to use six soil samples per ha, summing up to 24 samples per 4.17 ha field area. However this is not the optimal number of soil samples, which needs further study to confirm. The final matrix consisted of 146 samples collected from two fields in Vindumovergaard Farm Vindum, Denmark), 99 samples from Duck End farm (75 samples from Hawens End field and 24 samples from Ten Acre Meadow field; UK), 23 samples from one field in Ely Farm (Ely, UK), 12 samples from one field in Mespol Medlov, (Medlov, Czech Republic) and 14 samples from one field in Wageningen University experimental farm (Wageningen, The Netherland) [20,21]. The remaining 16 samples were used for validation of the on-line measurement. The former matrix was designated as calibration set, whereas the latter set was designated as validation set. The final matrix of the calibration set of 294 (24 + 270) was used to develop the pH calibration model.

The calibration spectra were subjected to spectra pre-treatment. Firstly, the noisy parts of the raw spectra at both edges were cut, which resulted in a final wavelength range of spectra of 400 to 2,100 nm. Secondly, soil spectra were subjected to wavelength reduction by averaging three and six neighbouring wavelengths in one wavelength in the ranges of 400–1,000 nm and 1,001–2,100 nm, respectively. This was followed successively by maximum normalisation, the first Savitsky-Golay derivation and smoothing with Savitsky-Golay method [9]. After spectra pre-treatment, partial least squares (PLS) regression with leave-one-out cross validation was carried out using the calibration set to develop the pH calibration model using Unscrambler 7.8 software (Camo Inc., Oslo, Norway).

The performance and accuracy of the pH calibration model was evaluated in cross-validation and independent validation. The independent validation was carried out using the spectra of the validation set of 16 soil samples. This was done using soil spectra collected under non-mobile conditions in the laboratory (off-line) and under mobile conditions during the on-line measurement. Model performance in cross-validation, on-line validation and off-line validation was evaluated by means of coefficient of determination (*R*^2^), root mean square error of prediction (RMSEP) and ratio of prediction deviation (RPD), which is standard deviation divided by RMSEP. Viscarra Rossel *et al.*[22] classified RPD values as follows: RPD < 1.0 indicates very poor model/predictions and their use is not recommended; RPD between 1.0 and 1.4 indicates poor model/predictions where only high and low values are distinguishable; RPD between 1.4 and 1.8 indicates fair model/predictions, which may be used for assessment and correlation; RPD values between 1.8 and 2.0 indicates good model/predictions where quantitative predictions are possible; RPD between 2.0 and 2.5 indicates very good, quantitative model/predictions, and RPD > 2.5 indicates excellent model/predictions. This classification system was adopted in this study. Sample statistics of the calibration and independent validation sets for pH model are shown in Table 2.

### 2.6. Development of Soil pH Maps

Two types of maps were developed; (1) pH and lime validation maps and (2) full-data points pH and lime recommendation maps. ArcGis 10 (ESRI, Redlands, CA, USA) software was used to generate the former maps, using inverse distance weighing (IDW) interpolation method. For these maps the 16 validation points were used. To produce the latter maps, Vesper 1.6 software, developed by Australian Centre for Precision Agriculture, was used to develop semivariogram models using the entire field on-line collected spectra. Based on semivariogram parameters and kriging interpolation method, ArcGis 10 was used to produce the full-data point map. The full-point maps were produced using the calibration dataset of 294 samples.

### 2.7. Development of Lime Recommendation Maps

The validation and full-point maps of pH were used to calculate the lime requirement. This was based on the 2010 fertilisation recommendation (RB209) of the UK Department for Environment, Food & Rural Affairs (DEFRA) [2], which requires pH in addition to texture as input data. Recommendations are usually calculated for the top 20 cm layer of the cultivated soil in arable lands or for the top 15 cm in grasslands. According to DEFRA [2] the lime recommendation for clay loams and clays of arable soils is 14 t/ha when pH is 5.0. For pH values of 5.5, 6 and 6.2, recommended lime applications are 10, 6 and 4 t/ha, respectively. Lime rate of 2 t/ha is recommended for pH values larger than 6.2.

## 3. Results and Discussion

### 3.1. Model Performance in Calibration and Independent Validation

**Figure 3.**
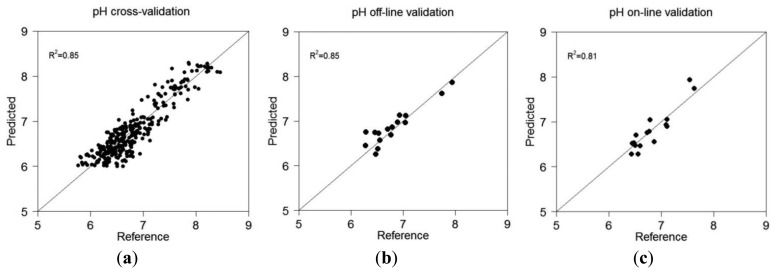
Scatter plot of predicted *versus* reference measured pH for (**a**) cross-validation, (**b**) off-line and (**c**) on-line validation sets.

When non-mobile collected soil spectra of the validation set were used for validation, this was designated as off-line validation, whereas this was designated as on-line validation when on-line collected spectra were used. Figure 3 shows the scatter plots of measured *versus* predicted pH in cross-validation, off-line validation and on-line validation.

Results show that pH calibration model in cross-validation results in very good accuracy (*R*^2^ = 0.85, RMSEP = 0.22 and RPD=2.43). This result over-performs those reported in the literature by [23] (Christy, 2008) (*R*^2^ = 0.62, RMSEP = 0.44) and [14] (Kodaira and Shibusawa, 2013) (*R*^2^ = 0.78, RMSEP = 0.19). The performance of the vis-NIR spectroscopy for the prediction of pH under off-line condition was better than that under on-line measurement condition. The off-line validation can be classified as excellent accuracy with *R*^2^ of 0.85, RMSEP of 0.18 and RPD value of 2.52 (Table 3), whereas the on-line prediction can be classified as very good with *R*^2^ of 0.81, RMSEP of 0.20 and RPD of 2.14 [22]. Shibusawa *et al.*[24] reported smaller accuracy with moderate prediction performance for on-line measurement with a smaller *R*^2^ of 0.62 and a larger RMSEP of 0.46.

The on-line validation results prove the on-line vis-NIR sensor [16] to provide very good measurement accuracy of soil pH. Not surprisingly, the off-line prediction accuracy is slightly better than the corresponding on-line, which might be attributed to other factors influencing the latter method [11]. These factors include among others noise associated with tractor vibration, sensor-to-soil distance variation, stones and plant roots and difficulties of matching the position of soil samples collected for validation with corresponding spectra collected from the same position [25].

### 3.2. Mapping

#### 3.2.1. Validation Maps of pH

**Figure 4.**
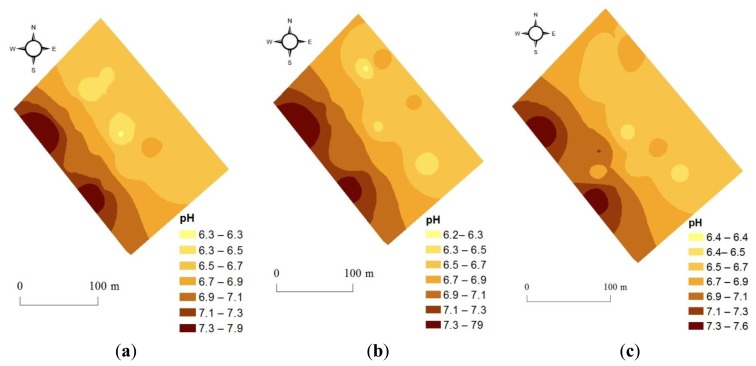
Comparison maps between (**a**) laboratory measured, (**b**) off-line visible end near infrared (vis-NIR) predicted, and (**c**) on-line vis-NIR predicted pH, based on 16 samples of the validation set.

Comparison between off-line and on-line predicted maps of the validation set (16 samples) show reasonable spatial similarity, with high and low zones matching well (Figure 4). This proves the high quality of on-line measured spectra, which is comparable to the quality of the corresponding spectra measured off-line in the laboratory. This is an indication of sensor stability and robustness during the on-line measurement.

#### 3.2.2. Full-Point pH Maps

Based on exponential semivariogram model, kriging on 2,160 on-line calculated soil pH values was performed to develop the full-point map of pH. Model parameters of the exponential semivariogram are listed in Table 4.

**Figure 5.**
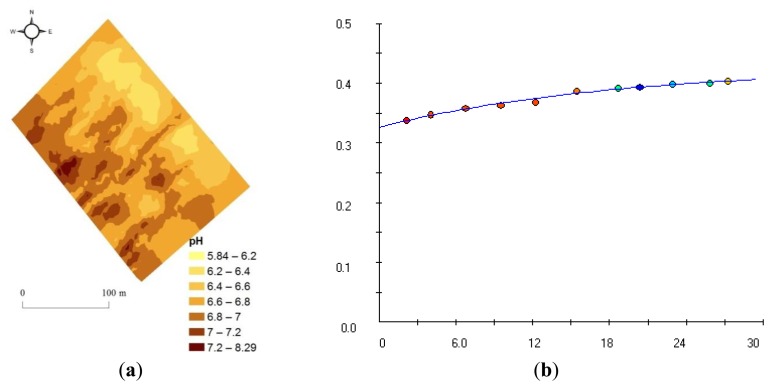
(**a**) Full-point pH map developed with kriging; (**b**) after exponential semivariogram.

This full-point pH map illustrates high spatial variability across the field area (Figure 5), ranging from 5.84 to 8.29. This high variability enhances the need for on-line soil sensor for the detailed characterisation of within field spatial variability of soil properties, as zones with different levels of concentration should be managed differently in precision agriculture [11]. Higher pH values are observed along the south-west side of the field, whereas lower concentrations can be observed on the opposite side of the field. In fact, this field can be divided into two clear parts of high and low pH concentration. This clear division can be explained by the fact that there is a drainage gutter on the south-west side of the field where soil is well drained and soil acidity has been lowered significantly.

#### 3.2.3. Lime Application Map

**Figure 6.**
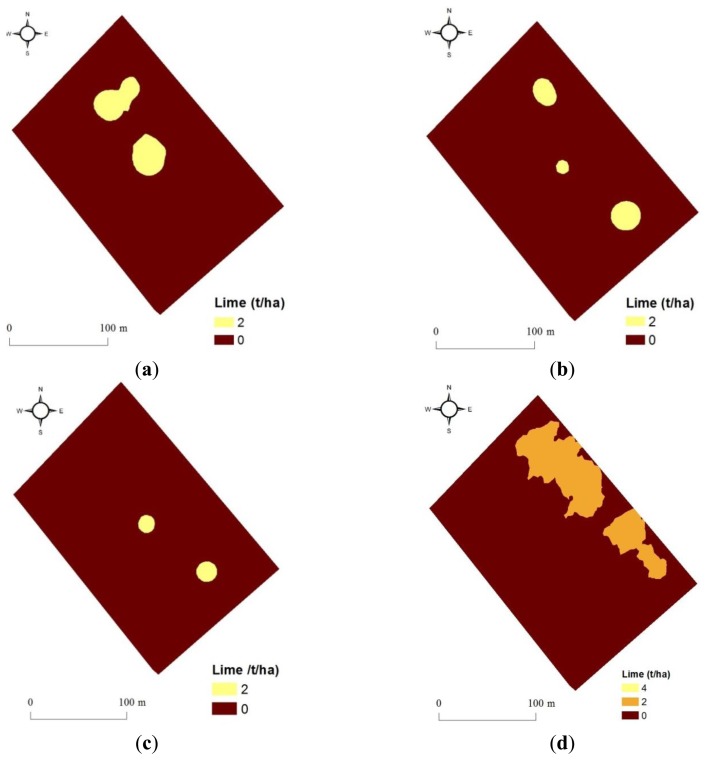
Lime application maps based on pH values obtained from (**a**) laboratory reference measured, (**b**) off-line visible and near infrared (vis-NIR) predicted and (**c**) on-line vis-NIR predicted, based on 16 validation points. (**d**) Lime recommendation map based on full-point on-line vis-NIR predicted pH values (2,160 points).

The average field pH value calculated for the 40 samples collected from the current field (Ten Acre Meadow) is 6.895. This value is too high (Table 5) to recommend any homogeneous lime application according to DEFRA RB209 fertilisation recommendation [2] (DEFRA, 2010). Variable rate lime application maps developed according to RB209 are shown in Figure 6. Recommendations based on a small number of samples (e.g., 16) of the validation set are generally of low rates and confirm that lime is needed with small amount only at 2-3 spots in the field. Comparing these three maps (Figure 6a–c) reveals partial spatial similarity, although the lowest total field application of 0.14 t is calculated with the on-line vis-NIR predicted pH map (Figure 6c and Table 5). Slightly higher total field applications of 0.28 and 0.48 t are calculated based on the off-line vis-NIR predicted (Figure 6b) and laboratory measured (Figure 6a) pH validation maps (16 points), respectively. However, lime rates recommended based on these three maps are negligible, and farmers may not apply any. Among all four maps, the recommendation based on on-line vis-NIR prediction with 2,160 points results in the highest lime application rate of 1.404 t (Figure 6c and Table 5). This considerable increase of application can be attributed to one of the following two reasons:

Firstly, it can be due to the insufficient degree of accuracy of pH prediction using on-line collected spectra. In fact, intervals between different pH categories used for the calculation of lime recommendations (DEFRA RB209) are very small (0.2–0.5). For example, for a small decrease in pH from 6.2 to 6, a large increase of lime from 4 to 6 t has to be recommended. However, the validation of pH calibration model using on-line spectra results in RMSEP of 0.20, which is equal to the difference between 6 and 6.2. This suggests that for deriving accurate lime recommendations based on on-line vis-NIR measurement of soil pH, RMSEP perhaps needs to be smaller than 0.20, which might necessitates the need for further refinement of pH prediction accuracy achieved so far in this study.

Secondly, the on-line vis-NIR sensor [16] provides detailed information about the spatial variation of soil properties due to the high resolution sampling rate of about 1,500–2,000 point/ha [12]. This might be the reason for the over-application of lime based on full-point pH map, as compared to the other three application maps with a limited number of pH reading of 16. If this hypothesis proves correct, then the on-line sensor has the potential of applying the correct amount of lime precisely in the right place.

Therefore, the vis-NIR spectroscopy can be successfully used for the prediction of soil pH and for deriving lime recommendations. Probably lime recommendations developed based on on-line full-point pH maps provide the highest precision of lime application. However, further research is needed in order to evaluate the influence of different lime recommendations on crop growth and yield, which will enable cost-benefit analysis. This is an essential requirement to arrive to a final conclusion on the potential of the on-line vis-NIR sensor for pH prediction and deriving meaningful lime recommendations.

## 4. Conclusions

This study was undertaken to map the spatial variability in soil pH in one clay field of 4.17 ha using an on-line vis-NIR soil sensor. Variable rate lime recommendation maps were developed under different sampling scenarios and these were compared to a corresponding lime map developed with laboratory reference measured pH. Results obtained led to the following conclusions:

1. Both off-line and on-line methods showed high prediction accuracy of pH with vis-NIR spectroscopy, which confirms the ability of vis-NIR spectroscopy to estimate pH having indirect spectral responses in the NIR range. However, a slightly higher accuracy was obtained with off-line (*R*^2^ = 0.85, RMSEP = 0.18 and RPD = 2.52), as compared to on-line (*R*^2^ = 0.81, RMSEP = 0.20 and RPD = 2.14) measured spectra of the validation set.
2. The full-point pH map demonstrated large spatial variability in soil pH, which was explained by the presence of a drainage gutter dividing the field into two almost equal parts.
3. Virtual calculation of lime requirement based on different pH sampling scenarios showed considerable differences between the developed recommendations. Recommendation based on on-line predicted full-point (e.g., 2,160) pH map required considerably higher lime rate (1.404 t), as compared to those developed based on a much smaller number of pH points (e.g., 16) for laboratory reference measurement (0.48 t), off-line prediction (0.28 t) and on-line prediction (0.14 t). A relatively larger error, or a much higher degree of detailed information about the spatial variation in pH obtained with the on-line measured full-point map might explain the higher lime rate. However, this might result in the most precise lime application in the field. Further research is needed in order to confirm this statement.

It is recommended to evaluate the agronomic and economic consequences of using the on-line vis-NIR sensor for deriving variable rate lime recommendation.

## Figures and Tables

**Table 1.** Information about the Ten Acre Meadow field in Duck End Farm, Bedfordshire, UK.

**Field**	**Area, ha**	**Crop**	**Sample Nr**	**Texture**	**Sand, %**	**Silt, %**	**Clay, %**	**Year**
Ten Acre Meadow	4.17	wheat	40	Clay	17.61	16.12	66.27	2012

**Table 2.** Sample statistics of laboratory measured pH of the calibration and independent validation sets.

**Calibration Set**					**Independent Validation Set**				

Nr.	Min	Max	Mean	SD	Nr.	Min	Max	Mean	SD *
294	5.84	8.29	6.82	0.58	16	5.99	7.62	6.75	0.43

* SD is standard deviation.

**Table 3.** Summary of pH model performance in cross-validation, off-line (laboratory) and on-line validations.

**Ten Acre Meadow Field**	***R*^2^**	RMSEP *	RPD *	**Intercept**	**Slope**
Cross-validation	0.85	0.22	2.43	0.88	0.86
Off-line independent validation	0.85	0.18	2.52	−0.26	1.02
On-line independent validation	0.81	0.20	2.14	−0.01	1.00

* RMSEP: root mean square error of prediction, RPD: ratio of prediction deviation.

**Table 4.** Parameters of the exponential semivariogram model used to develop full-point pH map.

**Property**	**Model fit**	**Nugget (C_0_)**	**Sill (C_0_+ C_1_)**	**Range**	**Proportion C_1_/C_0_+ C_1_**	**Sum of Square Error**
pH	exponential	0.3262	0.4262	18.25	0.2346	3.708

**Table 5.** Lime requirement calculated for different scenarios of pH estimation method.

**Soil pH**	**Lime t/ha**	**Laboratory Reference Measured (16 points)**		Off-Line vis-NIR * Predicted (16 points)		**On-Line vis-NIR Predicted (16 points)**		**On-Line vis-NIR Predicted (2,160 points)**	
		Area, ha	Lime, t	Area, ha	Lime, t	Area, ha	Lime, t	Area, ha	Lime, t
5	14	0	0	0	0	0	0	0	0
5.5	10	0	0	0	0	0	0	0	0
6	6	0	0	0	0	0	0	0	0
6.2	4	0	0	0	0	0	0	0.001	0.004
6.5	2	0.24	0.48	0.14	0.28	0.07	0.14	0.7	1.4
>6.5	0	3.93	0	4.03	0	4.10	0	2.346	0
**Total**		**4.17**	**0.48**	**4.17**	**0.28**	**4.17**	**0.14**	**4.17**	**1.404**

* vis-NIR is visible and near infrared.
